# Virtues of routine suprahyoid release during tracheal resection and anastomosis in patients with post intubation stenosis

**DOI:** 10.1007/s13304-024-02004-0

**Published:** 2024-09-30

**Authors:** Hany Hasan Elsayed, Ahmed Anwar El-Nori, Ahmed Mostafa, Mohamed Tarek Elsayegh, Samia Bassiouny, Ahmed Refaat, Mohamed Attia Elkahely, Mina Zaki

**Affiliations:** 1 Thoracic Surgery Department, Faculty of Medicine, Ain Shams University, Abbasia Square, Cairo, Egypt; 2 Cardiothoracic Surgery Department, Ain Shams University, Cairo, Egypt; 3 Department of otolaryngology, faculty of medicine, Ain Shams University, Cairo, Egypt; 4 Cardiothoracic surgery department, Faculty of medicine for girls, AlAzhar University, Cairo, Egypt

**Keywords:** Suprahyoid release, Tracheal resection, Dysphagia

## Abstract

Postintubation tracheal stenosis is the most common cause of benign tracheal stenosis. Surgical treatment is more challenging in long-segment stenosis. Suprahyoid release can increase tracheal length resected without anastomotic tension in patients with post-intubation tracheal stenosis. Its effect on swallowing has not been objectively studied and this article aims to explore its virtues and potential complications in a tertiary center for airway surgery. A prospective cohort study was conducted on forty consecutive patients from June 2020 till December 2023. Patients of both genders had tracheal resection anastomosis surgery with routine suprahyoid muscle release for resected tracheal segment of more than 2.5 cm in length aiming to decrease the anastomotic tension. Within two weeks postoperatively, a video naso-laryngoscope was done on all the patients to detect any vocal cord disorders, then they were examined by video fluoroscopy swallowing study VFSS to detect swallowing problems. Follow up was done for 6 months postoperatively. 40 patients were studied. Twenty-five patients (62.5%) were males. 21 patients (52.5%) had a cricotracheal resection. VFSS was performed on 38 patients (two patients excluded for serious morbidity). Six (15.7%) and four (10.5%) patients had residual semisolid and solid food in the vallecula and pyriform fossa respectively during swallowing. Five patients (13%) out of eight patients with abnormal VFSS had aspiration and dysphagia. Video nasolaryngoscopy was done pre- and post-operatively and showed that 7 patients (17.5%) had unilateral vocal cord paralysis, two of them had the same lesion preoperatively. Two patients developed postoperative anastomotic complications. All symptoms of dysphagia improved within 3 weeks of the procedure and improvement persisted for 6 months. Suprahyoid muscle release had a considerable reversible drawback on the process of swallowing. Its routine use in high-risk patients requiring long segment tracheal resections could be considered.

## Introduction

Advancement in the field of tracheal surgery with excellent outcomes occurred after understanding the historical background of this procedure and maneuvers to safely perform a well-vascularized low-tension anastomosis. In the past, it was believed that tracheal resection was better performed on a maximum length of 2 cm. In 1949, Rob and Bateman published a small series of patients in the British Journal of Surgery describing their experience with tracheal resection [1].

It was thought in the early twentieth century that cartilage healed poorly which made tracheal surgery poorly growing [2]. In 1898, Bruns did a lateral excision of a papillary tumour in the cervical trachea but managed the tracheal defect by packing and with a cannula [3]. By the mid-twentieth century, experiments confirmed that healing occurred following end-to-end anastomosis of both bronchi and trachea, although sometimes with stenosis [4, 5].

Baumann and Foster found that the human trachea could be stretched 4 to 6 cm by mobilization and that over half of the adult trachea could be resected and anastomosed by full mobilization of limiting structures [6] Mulliken and Grillo reported in 1968 an investigation of the length of the trachea that could be resected by cervical and mediastinal mobilization and still allow a safe anastomosis, leaving the pleural cavity intact [7].

One of the main dangers of airway surgery is complications related to anastomotic tension. A tension-free anastomosis is a goal for all tracheal surgeries. Hence release maneuvers were developed to allow drop of the larynx and reduction of anastomotic tension. Release maneuvers of the larynx and hyoid bone may be beneficial in patients with extended tracheal resections in upper and mid-tracheal lesions. Dedo and Fishman described the first laryngeal release between the thyroid cartilage and thyrohyoid membrane. This technique was complicated by post-operative dysphagia and aspiration [8], then Dedo moved away from this technique and described the suprahyoid muscle release maneuver that gave equivalent length of the trachea without risk of injury to superior laryngeal vessels and internal nerve branch, but the suprahyoid laryngeal release maneuver was first described in 1974 by Montgomery and was praised by decreasing the incidence of laryngeal dysfunction [9].

## Patients and methods

This prospective study included only adult patients who did tracheal resection anastomosis from June 2020 till December 2023 with a suprahyoid muscle release procedure. Our study excluded the patients in whom the suprahyoid release technique was not used and those who could not do videofluroscopy tests due to anastomotic dehiscence and inability to coordinate.

### Preoperative assessment

Descriptive data were collected including age, sex, duration of intubation, pre-operative tracheostomy, data regarding pre-operative naso videolaryngeoscopy, voice affection (aphonia or dysphonia), and degree of tracheal stenosis obtained from Virtual Bronchoscope. The level of the lesion was determined by the preoperative CT neck and chest with a virtual bronchoscope and confirmed by preoperative rigid and fiberoptic bronchoscopy and it was subglottic, tracheal, or both levels affected.

### Operative details

Type of the incision either collar, collar-manubriotomy or collar-sternotomy. Several removed tracheal rings and the length of the removed segment were also documented. Type of the operation is either cricotracheal resection, tracheal resection anastomosis, or laryngotracheal reconstruction.

### Suprahyoid release

The suprahyoid release is done through a low transverse collar incision, dissection is then carried down through subcutaneous fat and platysma muscle to the hyoid bone, the hyoid bone is exposed and dissection is carried out laterally to each side with care to preserve the digastric sling on either side, then the muscle attached to the hyoid bone is divided between the two slings of digastric muscles, these include mylohyoid, geniohyoid, genioglossus and tendons of chondroglossus muscle, the hyoid bone is divided on each side lateral to lesser cornu, but medial to digastric sling.

### Videoflurographic swallowing study VFSS

The videofluorographic swallowing study (VFSS), also known as a modified barium swallowing examination (MBS) is often considered the instrument of choice by the majority of practicing swallowing clinicians. Because it permits the visualization of bolus flow in relation to structural movement throughout the upper airway in real time. The VFSS also allows detection of the presence and timing of aspiration and assists in identifying the physiologic and often treatable cause of the aspiration [10, 11].

Capturing sequential video radiographic images of barium contrast added to food and liquid as they are transported during the oral cavity, pharyngeal cavity, and oesophagus in real-time. Radiographic images are used to administer various volumes and textures of food and liquid to patients and to obtain clinical impressions of the degree and presence of any swallowing impairment. Patients are informed and then undergo videofluoroscopy.

The protocol for this study involves patients chewing and swallowing thin fluids of varying sizes, starting with three cm of thin fluid to avoid aspiration, followed by five cm of thin fluids and finally a cup of drinking.

Semisolid foods were ingested and finally, solid foods were ingested. The images were obtained in anteroposterior and lateral views to allow detailed analysis of the patient's swallowing function.

Risks of aspiration are avoided by starting with a minimal amount of the material used during the test and observing any symptoms of choking.

Aspiration was defined as the passage of bolus material beyond the level of the true vocal cords, if material enters the larynx but remains above the vocal folds this is called penetration. To further evaluate the pharyngeal stage of the swallow, the presence or absence of swallow delay and reduced laryngeal elevation were obtained and the presence or absence of bolus residue along the base of the tongue, valleculae, piriform sinuses, posterior pharyngeal wall, and pharyngoesophageal segment were documented.

The data of the videofluroscopy test was documented and analyzed by the physician of phoniatrics and swallowing disorders. The pharyngeal phase of swallowing was studied in the following items: triggering of pharyngeal swallow, laryngeal elevation and epiglottic closure, residue in the vallecula and pyriform sinus, upper oesophagal release, presence of penetration and aspiration.

### Study procedure risks

We anticipate minimal additional risks by undertaking the study. The VFSS and videonasolarynoscope have minimal risks for the patients.

### Statistical analysis

Quantitative variables are expressed as mean ± standard deviation. While qualitative variables are expressed as frequency. Analysis was performed by statistical package software IBM-SPSS version 24.

### Ethical considerations

The study was approved by the Research Ethics Committee (REC) (MD 53/ 2020). Participants were given fully informed consent to participate in the study. These informed consents in this prospective study were written and verbal consents. Participant confidentiality and data security were guaranteed. Participants were able to withdraw from the research process at any time, were also able to withdraw their data if it was identifiable as theirs and were told when this would no longer be possible. Any expected benefits or potential harm to the research participant were thoroughly discussed.

## Results

### Preoperative data

The patients were 40 patients 25 (62.5%) were male and 15 (37.5%) females, the mean age was 38.1 ± 13.4 years, they were intubated for a period of 15.8 ± 2.61 days, there were 18 patients (45%) had a tracheostomy preoperatively.

Preoperative changes of voice recorded in our study, 20 patients (50%) had a normal voice, 7 patients (17.5%) had aphonia and 13 patients (32.5%) had dysphonia. Naso video laryngoscope was done on all the patients preoperatively and results were collected and showed 38 patients (95%) were normal and 2 patients (5%) had restricted mobility of the vocal fold. Level of lesion was documented and there was 1 patient (2.5%) isolated subglottic lesion, 18 patients (45%) isolated tracheal lesions and 21 patients (52.5%) lesions found in both subglottis and trachea.

### Operative data

The surgical approaches were 34 (84.5%) collar incision and 6 (15.5%) collar and manubriotiomy incision.

The length of the resected stenosed segment in cm was 4.6 ± 0.52 cm and the number of removed tracheal rings was 6.95 ± 1.11.

Operative techniques were 21 (52.5%) cricotracheal resection and 19 (47.5%) tracheal resection and anastomosis.

### Post-operative complications

Two patients developed anastomotic dehiscence and had a T tube insertion, and they did not complete the study. Those patients were old, ventilated and tracheostomized due to COVID-19 infection and they were on a prolonged course of steroids with high blood sugar. Both patients had cricotracheal resection and anastomosis. The resected segments were 3.1 and 3.7 cm respectively. They also had mediastinitis. One patient had his t-tube successfully removed and one patient required a permanent tracheostomy during the 6 months follow-up.

Complications were documented within two weeks for the patients, there were 26 patients (65.3%) with no complications, there were 3 patients (7.5%) and 5 patients (12.5%) who had superficial and deep wound infections respectively they were treated with antibiotics according to culture and sensitivity and cured. There were 2 patients (5%) who developed mediastinitis. There was no 30-day or 90-day hospital mortality in our study.

There were 5 patients (12.5%) who had symptoms of dysphagia to fluids and they were followed up by the nutrition specialist and were advised to ingest semisolid foods and drink water slowly, two patients of the five had the same symptoms preoperatively and their naso video- laryngoscope showed restricted mobility of one of the vocal folds. Those two patients were advised to follow-up at the otolaryngology clinic. The other 3 patients were followed up by calling them and they expressed improvement of their dysphagia symptoms within 3 weeks after surgery.

All preoperative, intraoperative, and postoperative data is shown in Table 1

**Table 1:** Descriptive, preoperative, operative characteristics and post-operative complications of the patients

Descriptive data	*n* (%)
Sex	
Female	15 (37.5%)
Male	25 (62.5%)
Level of the lesion	
Subglottic	1 (2.5%)
Tracheal	18 (45%)
Both	21 (52.5%)
Preoperative voice changes	
Normal	20 (50%)
Aphonia	7 (17.5%)
Dysphonia	13 (32.5%)
Preoperative naso videolaryngeoscope	
Normal	38 (95%)
Restricted mobility of vocal folds	2 (5%)
Type of operation	
Crico-tracheal resection	21 (52.5%)
Tracheal resection anastomosis	19 (47.5%)
Post operative naso videolaryngeoscope	
Normal	35 (87.5%)
Restricted mobility	5 (12.5%)
Post operative complications	
None	26 (65%)
Superficial wound infection	3 (7.5%)
Deep wound infection	5 (12.5%)
Mediastinitis	2 (5%)
Granulation tissue by bronchoscope	6 (15%)
Dysphagia	5 (12.5%)
Laryngeal edema	20 (50%)

Follow-up fiberoptic bronchoscopy was done on the 38 patients at the endoscopy unit of the hospital within one month after the operation. Results showed that 6 patients (15.7%) had mild granulation tissues at the suture line, and they did not need any dilatation procedures and were advised to visit our clinic after one month for follow-up. No patient required redo surgery or tracheostomy insertion. We had 20 (50%) of patients with laryngeal edema.

### Videofluroscopy Swallowing Study (VFSS)

**Fig. 1:**
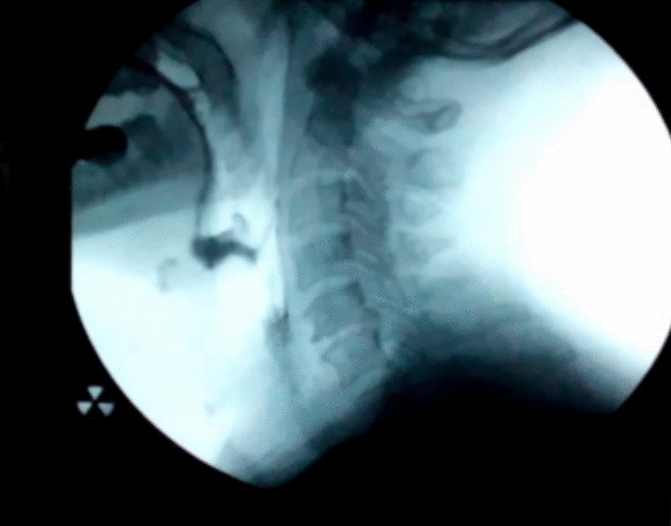
Video fluoroscopy image showing semisolid residue in the vallecula and pyriform fossa

The results of the video fluorographic swallowing study (VFSS) revealed that all 38 patients had normal triggering of pharyngeal swallow, laryngeal elevation and epiglottic closure. There were 6 patients (15.7%) with semisolid residue in the vallecula and pyriform sinus (Fig. 1), four patients emptied the residue after 2 swallows and 2 patients after 1 swallow, no patient had dysphagia due to this only finding.

The presence of penetration or aspiration of the bolus was observed.

Eight patients had an abnormal VFSS (4 from the CTRA group and 4 from the TRA group). Only five out of eight were symptomatic (Table 2).

**Table 2:** shows the results of the VFSS study

40 patients had suprahyoid release maneuver					
38 patients were eligible to VFSS					
Result of the study = Number of patients	Normal study = 30	Abnormal study = 8			
		Semisolid residue = 2 (1 CTRA and 1 TRA)	Solid residue = 1 (TRA)	Combined penetration aspiration = 1 (TRA)	Combined semisolid residue, penetration and aspiration = 4 (3 CTRA and 1 TRA)
Symptoms	Asymptomatic	Asymptomatic	Asymptomatic	Dysphagia	Dysphagia
Follow up	No follow-up up needed			The patient had improvement of symptoms within 6 months of follow-up	

*CRTA* crico-tracheal resection and anastomosis, *TRA* tracheal resection and anastomosis

There were no patients with penetration to semisolid or solid boluses, but there were 5 patients (13.1%) with penetration to liquids. Those patients had dysphagia and aspiration was found also in the 5 patients.

Three patients had an abnormal VFSS with no symptoms of dysphagia.

### Follow-up

The five symptomatic patients were followed up and symptoms disappeared within 3 weeks and improvement persisted for 6 months postoperatively.

## Discussion

To the best of our knowledge, this is the first study to objectively investigate the effect of routine suprahyoid muscle release maneuvers on deglutition in patients performing crico-tracheal/tracheal resection and anastomosis for post-intubation tracheal stenosis.

The surgeon’s decision to preform a suprahyoid release is weighed between not performing it increasing the risk of extra tension on the anastomosis especially in long segment tracheal resection and the risk of postoperative dysphagia if performed which may be unnecessary, especially in short segment resection. This study may help to subjectively and objectively highlight the relative safety of suprahyoid release which may encourage airway surgeons to have a low threshold of performing the procedure if suspicion of anastomotic tension is high.

Human cadaveric studies have shown that the tension increases exponentially with the length of the stenosis resected [12, 13]. Without release techniques, a cervical tracheal defect of 2 cm or less can be managed by primary anastomosis [13]. Consequently, in our study, we chose a routine suprahyoid release for all patients requiring 2.5cm of tracheal or more to reduce anastomotic tension. Previous authors had quantified the longest tracheal defect feasibly resectable using their specific muscle release: suprahyoid claiming up to 5 cm [9] and infrahyoid claiming up to 4 cm [8].

The degree of release obtained as well as the inherent elasticity of the trachea vary greatly among patients and depend greatly on age and previous surgery.

Critics of the infrahyoid release technique cite an increase in postoperative dysphagia. Grillo wrote that aspiration was commonly seen early after thyrohyoid membrane and infrahyoid muscle release and less often after suprahyoid muscle release [14]. We believe that a suprahyoid muscle release may be necessary for tension-free closure in cases of longer tracheal resections and prefer it as a laryngeal release maneuver for the same reason.

Komori et al. [15] documented that they operated on six patients with severe long-segment congenital tracheal stenosis using supra hyoid muscle release and mentioned that patients experienced no impaired deglutition problems nor recurrent laryngeal nerve injury. Obviously, this was a subjective and small study, but more objective evidence can highlight abnormal deglutition after suprahyoid release.

Video fluoroscopy study (VFSS) has become the accepted standard for the evaluation of swallowing. Hence, we used it as a reliable examination of swallowing as all VFSS studies include image sequences that can be digitized and analyzed using various software applications. These techniques make measurements more precise by allowing frame-by-frame analysis, thereby increasing intra- and inter-rater reliability [16, 17].

Many surgeons believe that the suprahyoid release is an effective way to resect the upper portion of the trachea in cervical lesions. In our current study, all the cervical lesion patients received a suprahyoid release maneuver if the tracheal segment required to be resected was more than 25mm. Not all institutes will practice routine suprahyoid release. The Massachusetts General Hospital reported that only 46 out of 521 tracheal resection surgeries for post-intubation stenosis involved a laryngeal suprahyoid release. The incidence of laryngeal suprahyoid release increased from 6.4% in primary operations to 29% in re-operations. Satisfactory results were reported for these patients [18].

In another series of laryngotracheoplasty resections reported by the same institute, laryngeal release manoeuvres were performed in only 8.7% of 80 patients [19]. After suprahyoid release, the most commonly reported symptoms in their series were dysphagia and aspiration, similarly to what we reported.

Our study found that 5 patients had evidence of restricted mobility of the vocal folds. Two of them were found preoperatively, but for the other 3 patients we thought that it was due to paresis of the recurrent laryngeal nerve, this required a follow-up naso video laryngoscope.

Our study has shown that VFSS is an excellent negative test for swallowing assessment after suprahyoid release in the case of CTRA/TRA as all patients report postoperative dysphagia. However, only 5 patients out of 8 with an abnormal VFSS were symptomatic. It appears that VFSS is sensitive to diagnose mild cases of swallowing abnormalities not reported by patients.

In our study, 26 patients (65%) were free of any complications. Complications were mostly non-anastomotic complications. We supposed that there was a higher incidence of wound infection among our patients due to previous history of COVID-19 infection and steroid treatment for a long time. Patients who developed tracheal anastomotic dehiscence were excluded and did not complete the VFSS study, because of their bad general condition, long hospital stay and difficulty to do swallowing investigations for them.

Besides the maneuvers described in our study, longer trachea resection with more complex release maneuvers is infrequently needed. These release maneuvers are especially recommended for re-do trachea resections and longer stenosis and tumor involvements. These operations are generally performed in our institution with a median sternotomy or clamshell incision. Extensive Hilar release maneuvers are not only performed to release the superior and inferior pulmonary vein but also include the pulmonary arteries. These include the division of broncho-pericardial and tracheo-pericardial ligaments. Another important release maneuver is pericardiophrenic release which involves dissection of inferior pericardium from the diaphragm. These release maneuvers are reported to be important in extensive resections (20).

The limitations of our study include being a single institution study, lack of a control group and not repeating VFSS in patients who had an abnormal study and depending on follow-up of symptoms which showed improvement in all cases.

## Conclusion

Suprahyoid muscle release had a considerable reversible drawback on the process of swallowing. Its routine use in high-risk patients requiring long segment tracheal resections could be considered.

## Data Availability

Available.

## Abbreviations

VFSS: Video fluorographic swallowing study
MBS: Modified barium swallowing examination
TIF: Tracheoinnominate fistula
CT: Computed tomography
N: Number
CRTA: Crico tracheal resection and anastomosis
TRA: Tracheal resection and anastomosis
